# Supplementary material for: Population Pharmacokinetics of Amikacin in Patients on Veno-Arterial Extracorporeal Membrane Oxygenation
Source: Pharmaceutics. 2022 Jan 26;14(2):289. doi: 10.3390/pharmaceutics14020289 (PMC8879580; doi:10.3390/pharmaceutics14020289)
Supplement: Supplementary file 1 [file pharmaceutics-14-00289-s001.zip › pharmaceutics-1541524-supplementary.pdf]

pharmaceutics

MDPI

Supplementary Materials: Population Pharmacokinetics of Amikacin in Patients on Veno-Arterial Extracorporeal Membrane Oxygenation

Claire Pressiat, Agathe Kudela, Quentin De Roux, Nihel Khoudour, Claire Alessandri, Hakim Haouache, Dominique Vodovar, Paul-Louis Woerther, Alice Hutin, Bijan Ghaleh, Anne Hulin, Nicolas Mongardon

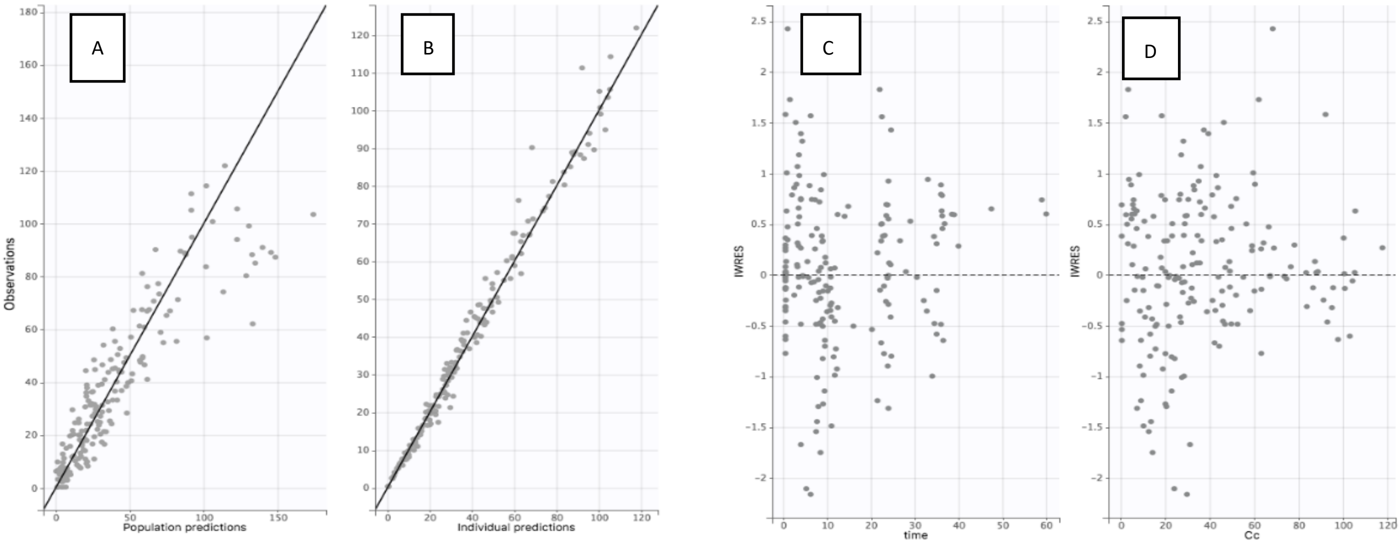

Figure S1. Diagnostics plots of final model: (**A**). Observed concentrations *vs* predicted concentrations of populations; (**B**). Observed concentrations *vs* individual predicted concentrations; (**C**). Individual weighted residues (IWRES) in time; (**D**). Individual weighted residues (IWRES) *vs* observed concentrations (Cc).

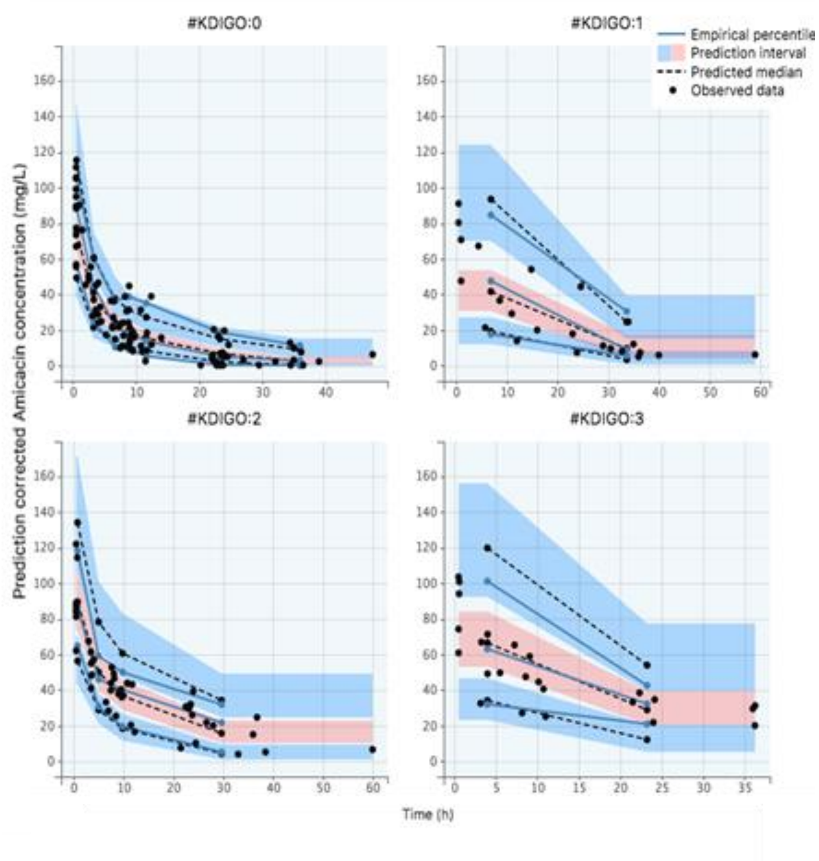
#KDIGO:0
#KDIGO:1
#KDIGO:2
#KDIGO:3
Empirical percentile
Prediction interval
Predicted median
Observed data
Prediction corrected Amicacin concentration (mg/L)
Time (h)

(**A**)

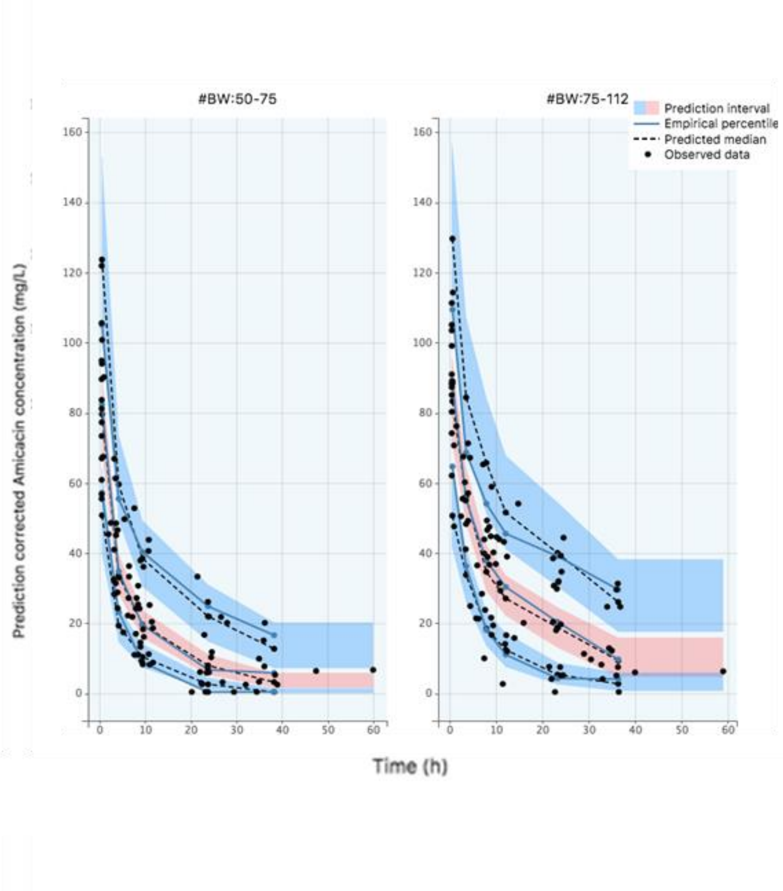
#BW:50-75
#BW:75-112
Prediction interval
Empirical percentile
Predicted median
Observed data
Prediction corrected Amicacin concentration (mg/L)
Time (h)

(**B**)

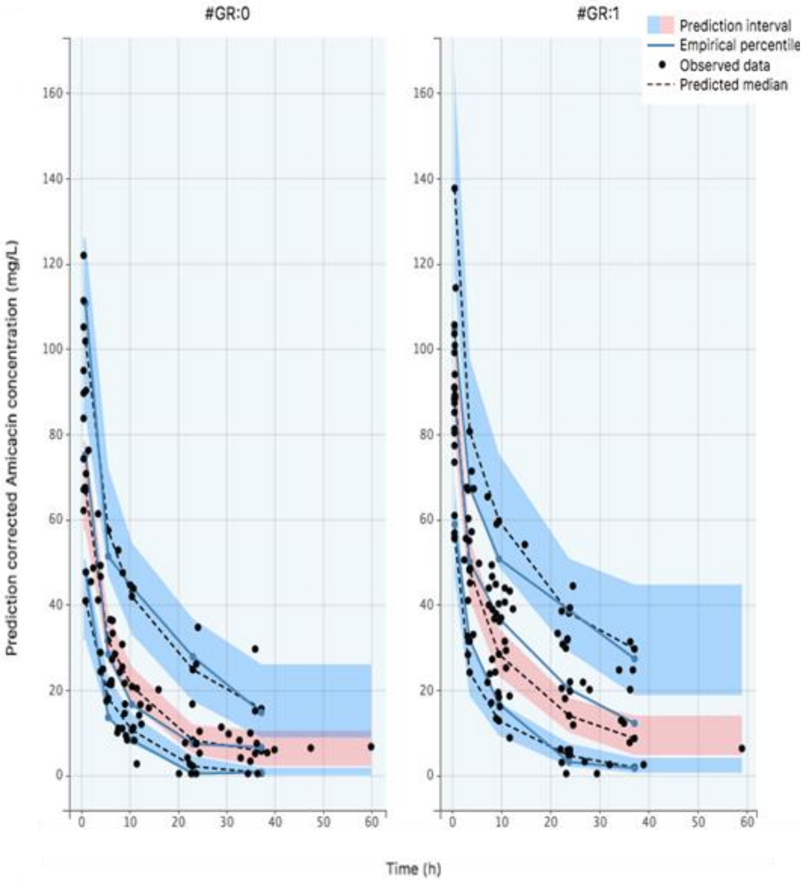

(**C**)

Figure S2. PC-VPC of final model: Depending on the KDIGO stage (**A**). Depending on the TBW range (**B**). Depending on the absence or presence of V-A ECMO support (GR = 0 for control, GR = 1 for V-A ECMO) (**C**).
